# Early Detection of A*β* Deposition in the 5xFAD Mouse by Amyloid PET

**DOI:** 10.1155/2018/5272014

**Published:** 2018-02-28

**Authors:** Se Jong Oh, Hae-June Lee, Kyung Jun Kang, Sang Jin Han, Yong Jin Lee, Kyo Chul Lee, Sang Moo Lim, Dae Yoon Chi, Kyeong Min Kim, Ji-Ae Park, Jae Yong Choi

**Affiliations:** ^1^Division of RI-Convergence Research, Korea Institute of Radiological and Medical Sciences, Seoul, Republic of Korea; ^2^Radiological & Medico-Oncological Sciences, University of Science and Technology, Daejeon, Republic of Korea; ^3^Division of Basic Radiation Bioscience, Korea Institute of Radiological and Medical Sciences, Seoul, Republic of Korea; ^4^Department of Nuclear Medicine, Korea Institute of Radiological and Medical Sciences, Seoul, Republic of Korea; ^5^Research Institute of Labeling, FutureChem Co., Ltd., Seoul, Republic of Korea; ^6^Division of Medical Radiation Equipment, Korea Institute of Radiological and Medical Sciences, Seoul, Republic of Korea

## Abstract

*Purpose.* ^18^F-FC119S is a positron emission tomography (PET) tracer for imaging *β*-amyloid (A*β*) plaques in Alzheimer's disease (AD). The aim of this study is to evaluate the efficacy of ^18^F-FC119S in quantitating A*β* deposition in a mouse model of early amyloid deposition (5xFAD) by PET.* Method*. Dynamic ^18^F-FC119S PET images were obtained in 5xFAD (*n* = 5) and wild-type (WT) mice (*n* = 7). The brain PET images were spatially normalized to the M. Mirrione T2-weighted mouse brain MR template, and the volumes of interest were then automatically drawn on the cortex, hippocampus, thalamus, and cerebellum. The specific binding of ^18^F-FC119S to A*β* was quantified as the distribution volume ratio using Logan graphical analysis with the cerebellum as a reference tissue. The A*β* levels in the brain were also confirmed by immunohistochemical analysis.* Result*. For the 5xFAD group, radioactivity levels in the cortex, the hippocampus, and the thalamus were higher than those for the WT group. In these regions, specific binding was approximately 1.2-fold higher in 5xFAD mice than in WT. Immunohistochemistry supported these findings; the 5xFAD showed severe A*β* deposition in the cortex and hippocampus in contrast to the WT group.* Conclusion*. These results demonstrated that ^18^F-FC119S PET can successfully distinguish A*β* depositions in 5xFAD mice from WT.

## 1. Introduction

Alzheimer's disease (AD), the most common neurodegenerative disorder, is characterized by cognitive and memory deterioration as a consequence of abnormal deposition of amyloid-beta (A*β*) and neurofibrillary tangles of hyperphosphorylated tau [1–3].

Early diagnosis of AD, before the advent of structural changes, is important to reduce the socioeconomic burden of the disease. Molecular imaging with positron emission tomography (PET) is a promising tool because it can functionally detect the burden of A*β* plaques and evaluate longitudinal changes in patients with AD [4, 5]. Radiochemists designed many other A*β*-targeted PET radiotracers in an effort to satisfy one with more drug-like properties. Klunk et al. developed ^11^C-Pittsburgh compound B (PIB), which has high specificity and affinity for A*β* [6]. However, this radiopharmaceutical has an intrinsic limitation for multicenter studies. ^11^C has a short half-life of only 20 min, and thus, ^11^C-based radiotracers can be used only at a center with an on-site synthetic facility [7]. Later, ^18^F-florbetapir, ^18^F-flutemetamol, and ^18^F-florbetaben, which detect the presence of A*β* plaques, were developed and approved by the US Food and Drug Administration [8–10]. Previous PET studies have shown that they have both succeeded and failed in detecting amyloidosis in AD animal models [11–15]. Recently, ^18^F-FC119S (2-[2-(N-monomethyl)aminopyridine-6-yl]-6-[(S)-3-fluoro-2-hydroxypropoxy]benzothazole) is developed A*β* imaging PET tracer. This tracer has high selectivity and metabolic stability against troublesome* in vivo* defluorination [16]. In addition, ^18^F-FC119S showed a greater binding value in the APP/PS1 than their wild-type (WT) counterparts [17]. In the first in human study including patients with mild cognitive impairment and AD, ^18^F-FC119S displays significant linear correlation with ^11^C-PIB [18].

Several transgenic mouse lines have been used for amyloid PET research, but only a limited range of AD mice can be used [14, 15]. The 5xFAD mouse is a transgenic model of AD carrying five mutations associated with early onset familial Alzheimer's disease (FAD): the K670N/M671L (Swedish), I716V (Florida), and V717I (London) mutations in human amyloid precursor protein (APP) and the M146L and L286V mutations in human presenilin-1 (PS1) [19–22]. All five FAD mutations synergistically promote the occurrence of A*β*_42_; therefore, this model shows very aggressive A*β* deposition in the brain starting at 1.5 months of age [23].

The purpose of the present study is to examine the efficacy of ^18^F-FC119S PET for distinguishing A*β* deposition in an AD mouse model of early amyloid deposition from WT.

## 2. Materials and Methods

### 2.1. Animals

Two groups of mice were used for these studies: 5xFAD mice (male, *n* = 5) and WT counterparts (male, *n* = 7), all at the age of 5.5 months. The care, maintenance, and treatment of animals in these studies followed protocols approved by the Institutional Animal Care and Use Committee of Korea Institute of Radiological & Medical Sciences (KIRAMS), and the experiments involving animals were performed according to the Guide for the Care and Use of Laboratory Animals published by the US National Institutes of Health. The animal housing room was automatically controlled with a temperature of 22 ± 3°C, a 12 h/12 h light/dark cycle and 55 ± 20% humidity. Sterilized rodent diet and purified tap water were supplied ad libitum.

### 2.2. Preparation of ^18^F-FC119S

^18^F-FC119S was synthesized by nucleophilic substitution of F-18 on the precursor as previously described in Byun et al.'s work [18]. The specific activity of ^18^F-FC119S was greater than 44 GBq/*μ*mol, and the mean radiochemical purity was 99% (*n* = 4).

### 2.3. PET/CT Scan

PET images of the mice were obtained using a small-animal PET scanner (nanoScan®, Mediso, Budapest, Hungary), consisting of two rings with 12 PET detector modules. The spatial resolution at 1 mm off the center of the scanner in the axial, radial, tangential directions is 0.85, 0.8, and 0.8 mm, respectively. Mice were anesthetized with 2.5% isoflurane, and ^18^F-FC119S (8.9 ± 1.3 MBq/200 uL) was injected through the tail vein with a syringe pump (KDS 210, KD Scientific, Holliston, MA) over the course of 1 min. Simultaneously, dynamic PET scanning was performed for 60 min. Images were reconstructed with user defined time frames (14 × 30 s, 3 × 60 s, 4 × 300 s, 3 × 600 s, 24 frames in total) using a 3-dimensional ordered subset expectation maximization algorithm. For attenuation correction and anatomical reference, a computed tomography (CT) scan was acquired immediately after PET (50 kVp of X-ray voltage at 0.16 mAs).

### 2.4. Image Analysis

**Figure 1:**
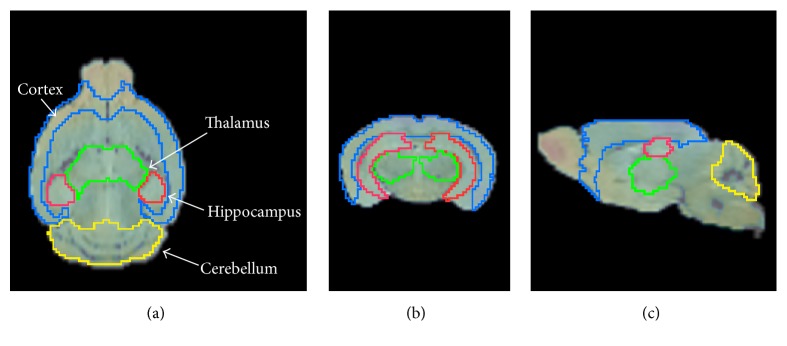
Definition of VOIs from a 5xFAD mouse in the horizontal (a), coronal (b), and sagittal (c) planes. The PET was spatially normalized to the T2-weighted mouse brain MR template.

According to Oakley et al., A*β* deposition begins to appear in the cortex, the hippocampus and the thalamus of 5xFAD mice at 1.5 months of age [23]. However, no significant increase in A*β* was found in the olfactory bulbs, striatum, hypothalamus, or thalamus at 5 months of age [24]. Therefore, we selected the cortex, hippocampus, and the thalamus as volumes of interest (VOIs). To create a study-specific PET brain template, dynamic PET images were motion-corrected and spatially normalized to the T2-weighted mouse brain MR template (M. Mirrione, embedded PMOD software). Four VOIs were defined on the MRI template; cortex (149.2 mm^3^), hippocampus (24.9 mm^3^), thalamus (28.2 mm^3^), and cerebellum (57.2 mm^3^, Figure 1). Finally, regional time-activity curves (TACs) were generated. The obtained uptake value was determined for each brain region and presented as a standardized uptake value (SUV). The SUVs were obtained by normalizing tissue radioactivity concentration to injected dose and body weight [25].

To compare the specific binding of ^18^F-FC119S to A*β* deposits, we used Logan graphical analysis (*t*^*∗*^ = 10 min) to derive a distribution volume ratio (DVR) based on the cerebellum [26]. All image analysis was conducted with PMOD (version 3.4, PMOD Group, Graubunden, Switzerland).

### 2.5. Immunohistochemistry

The animals were euthanized, and brain samples were fixed in 4% paraformaldehyde for 48 hours, embedded in paraffin, and sectioned at 5 *μ*m intervals. Immunohistochemistry was conducted using the Vectastain Elite ABC kit (Vector Laboratories Inc., Burlingame, CA, USA) following the manufacturer's protocol. For antigen retrieval, the sections were placed in a citrate buffer (pH 6.0) and heated in boiling water for 30 minutes. The sections were then placed in 0.3% H_2_O_2_ in absolute methanol for 15 minutes at room temperature to block the endogenous peroxidase. Sections were then incubated overnight at 4°C with mouse anti-6E10 antibody (1 : 1000, SIG-39320, Covance, Emeryville, CA), washed, and incubated with the corresponding secondary antibody. ImageJ was used to quantify the amount of A*β* in each brain section. As a control, the primary antibody was omitted from several test sections in each experiment. The sections were counterstained with Harris' hematoxylin prior to mounting.

For analysis of the amyloid plaques, the ImageJ software (https://rsbweb.nih.gov/ij/) was used. After adjusting for threshold, ImageJ was used to measure total area of the plaques and the percentage of total brain area occupied by plaques. The brain area (cortex or hippocampus) was outlined in the right hemisphere using the edit plane function. Data were pooled from 8~12 sections at ×200 magnification of each mouse and 5 mice were used for the statistical analysis.

### 2.6. Statistical Analysis

The quantitative results are expressed as the mean ± SD. All statistical results were analyzed with Prism (GraphPad Software, Inc., CA). Student's *t*-test was used to determine statistical significance at the 95% confidence level, with *p* < 0.05 indicating a significant difference.

## 3. Results

### 3.1. ^18^F-FC119S PET Images

**Figure 2:**
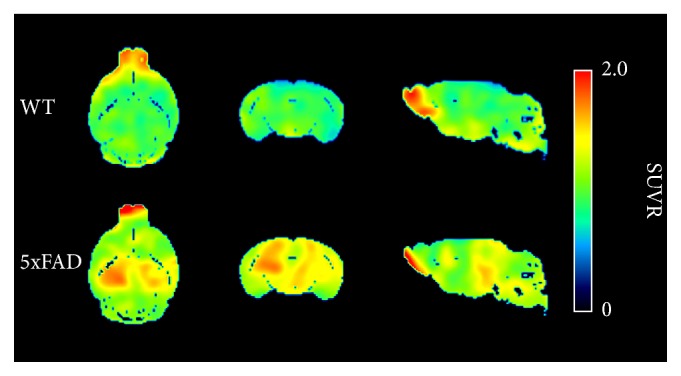
^18^F-FC119S summed PET images (30–60 min) of the WT and the 5xFAD in the one representative animal. The color scale indicates the SUV ratio.

The ability of ^18^F-FC119S to quantify A*β* burden was assessed by PET. PET images showing the brain SUV ratio (30–60 min) are shown in Figure 2. By visual inspection, the 5xFAD showed higher cortical and hippocampal ^18^F-FC119S uptake than the WT group. Radioactivity was also detected in the olfactory bulb in both groups, but this uptake was due to spill-over from the Harderian gland [27].

**Figure 3:**
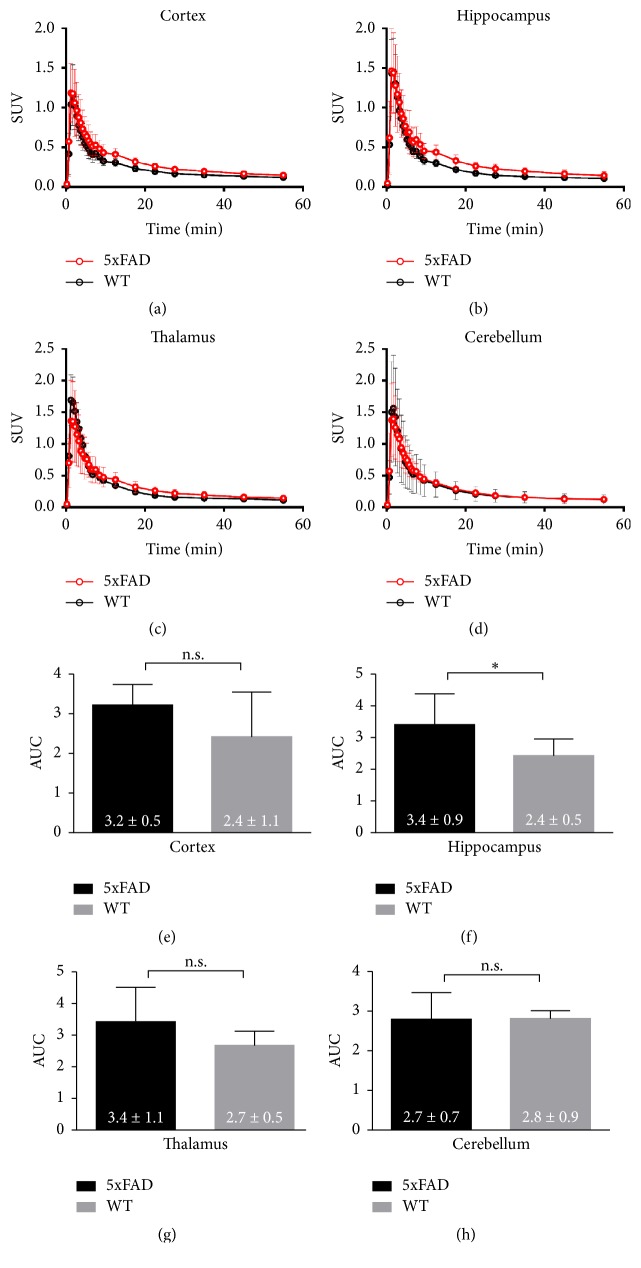
Time-activity curves of the cortex (a), hippocampus (b), thalamus (c), and cerebellum (d). Area under the curve (AUC) for the cortex (e), hippocampus (f), thalamus (g), and cerebellum (h). Data are presented as the mean ± SD. (^*∗*^*p* < 0.05, n.s. = statistically nonsignificant difference).

Figure 3 represents the regional TACs. After approximately 10 minutes, the cortex and hippocampus and the thalamus of the 5xFAD showed greater uptake than those of the WT (Figures 3(a)–3(c)). In contrast, radioactivities in the cerebellum, which we used as the reference region, did not differ between groups (Figure 3(d)). Area under the curve (AUC) values was obtained from 30 to 60 minutes by the trapezoid rule (Figures 3(e)–3(h)). In 5xFAD mice, the AUC values of the cortex, hippocampus, and thalamus showed 33.3, 41.7, and 25.9% increments, respectively, compared with the corresponding values in WT mice. Among VOIs, only hippocampus showed statistically significant difference (*p* = 0.0479). The difference of cerebellar uptakes between 5xFAD and WT was not statistically significant (*p* = 0.9299).

### 3.2. Distribution Volume Ratios

**Figure 4:**
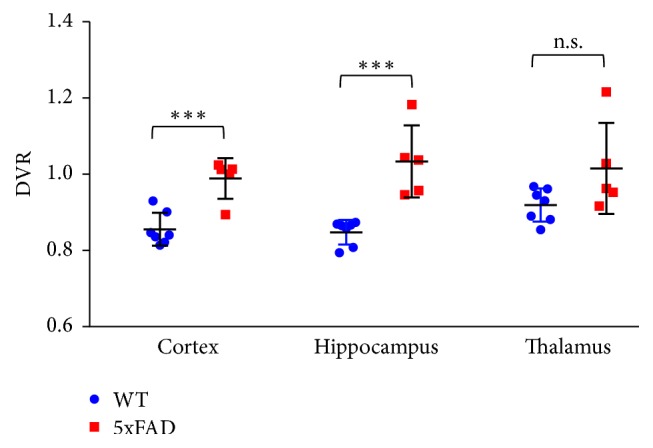
The DVR values according to Logan graphical analysis. Statistical significance was defined as a *p* value less than 0.05 (^*∗∗∗*^*p* < 0.001, n.s. = statistically nonsignificant difference).

To elucidate the specific binding level of ^18^F-FC119S, we calculated the DVR values (Figure 4). The mean DVR values for the 5xFAD were 10–21% higher than those for the WT group (DVR values in cortex: 5xFAD = 0.99 ± 0.05 versus WT = 0.86 ± 0.04, *p* = 0.0007; in hippocampus: 5xFAD = 1.03 ± 0.09 versus WT = 0.85 ± 0.03, *p* = 0.0006; in thalamus: 5xFAD = 1.01 ± 0.12 versus WT = 0.92 ± 0.04, *p* = 0.0748).

### 3.3. Immunohistochemical Staining

**Figure 5:**
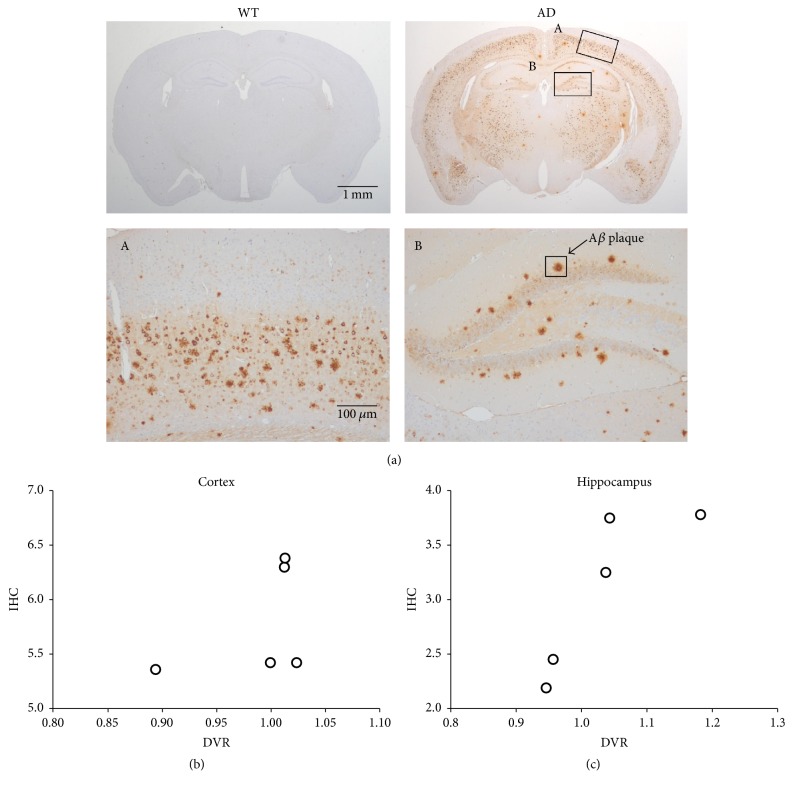
Immunohistochemical staining of A*β* in the brains of WT and 5xFAD mice (a). Insets represent high-magnification images of the cortex (A) and hippocampus (B). Relationship between IHC data and DVRs of cortex (b) and hippocampus (c).

To assess the actual A*β* burden in the brain, we performed immunohistochemistry (Figure 5). While A*β* deposition was not detected in the WT mice, increased A*β* burden was identified in 5xFAD (Figure 5(a)). The quantification revealed that the A*β* deposition was greater in the cortex than in the hippocampus (Figure S1, 6.13 ± 0.76% and 3.08 ± 0.73%, resp.). These results were consistent with previous studies showing that A*β* filled most of the hippocampus and cortex of 5xFAD mice within 6 months [28]. However, A*β* plaques were not expressed in both 5xFAD and WT (Figure S2). The association between A*β* deposition and DVRs showed that the cortex has low correlation (Figure 5(b), *r* = 0.45) whereas the hippocampus has strong correlation (Figure 5(c), *r* = 0.85).

## 4. Discussion

To the best of our knowledge, the present study is the first to demonstrate that ^18^F-FC119S PET can successfully differentiate the A*β* burden of 5xFAD mice from that of WT mice. These PET results were validated by immunohistochemistry. From this perspective, ^18^F-FC119S may be a useful PET radiotracer for A*β* imaging.

Pathologically, A*β* deposition in the brain occurs first in the cortical area (6–9 months), followed by the hippocampus (12–15 months) [29–32]. This implies that A*β* plaques have a relatively lower concentration in the limbic system than in the cortex at a young age [30]. Previous rodent amyloid PET studies using ^11^C-PIB or ^18^F-florbetaben failed to discriminate between young AD mice (PS2APP, G384A, APP/PS1; all mice age less than 12 months) and WT mice in terms of specific binding in the hippocampus [15], whereas the differences of binding values in 20-month-aged APP/PS1 mice were successfully detected by ^18^F-FC119S [17]. Rojas et al. also demonstrated that increased cerebral uptakes in 10–16-month-aged 5xFAD by ^18^F-florbetapir [12]. In addition to genetic modification, age may be a critical factor. Pathological changes in the hippocampus are observable in advanced AD mice. Longitudinal PET studies must be conducted for 20 months or longer to detect these changes [33]. However, this long timeframe makes it difficult to choose an appropriate age for analysis and to maintain the animals in good condition for the entire study. 5xFAD line displays high concentration of A*β* as well as cognitive dysfunction around 4 months of age. Moreover, these mice develop neuron loss, unlike most other hAPP and hAPP/PS1 models [34]. Regarding this issue, 5xFAD mice may offer an advantage over other transgenic mice. Due to the aggressive A*β* deposition in this model, we confirmed the presence of detectable changes even at the age of 5.5 months. The benefit of 5xFAD model is the early amyloid deposition, which could offer to longitudinal experiments over APP/PS1.

The current PET results are an underestimate of the in vitro values. In our immunohistochemistry analysis, the cortical and hippocampal A*β* plaque concentration was evidently increased in the 5xFAD, while no A*β* was detected in WT mice. Despite this in vitro result, the difference between binding values was only 16–22%. These patterns are consistent with previous studies using APP/PS1 transgenic mice [17, 19]. This observation may be related to experimental techniques. Dynamic PET images provide information on the pharmacokinetics and pharmacodynamics of the tracer in the body [35]. On the other hand, histological examination reflects antigen-antibody binding ability and is observed in a brain slice under static incubation conditions. Partial volume effect by the small brain structure and limited PET resolution also attribute the underestimation of PET signal.

## 5. Conclusion

In summary,^ 18^F-FC119S PET showed selective binding to pathological A*β* deposits in 5xFAD mice. The cortical and hippocampal uptake of the tracer was higher in the 5xFAD than in WT mice. These results were reproduced by immunohistochemistry. Therefore, ^18^F-FC119S would be a suitable candidate preclinical PET radiotracer.

## Supplementary Materials

Supplementary Materials Figure S1: quantification of A*β* deposition in the cortex and hippocampus. Values are presented as the mean ± SD; n.d.: not detected. Figure S2: immunohistochemical staining of A*β* in the cerebellum of WT and 5xFAD mice.

## References

[1] Poisnel G., Hérard A.-S., El Tannir El Tayara N. (2012). Increased regional cerebral glucose uptake in an APP/PS1 model of Alzheimer's disease. *Neurobiology of Aging*.

[2] Villemagne V. L., Pike K. E., Darby D. (2008). A*β* deposits in older non-demented individuals with cognitive decline are indicative of preclinical Alzheimer's disease. *Neuropsychologia*.

[3] Selkoe D. J. (2001). Alzheimer's disease: genes, proteins, and therapy. *Physiological Reviews*.

[4] Nordberg A. (2004). PET imaging of amyloid in Alzheimer's disease. *The Lancet Neurology*.

[5] Johnson K. A., Fox N. C., Sperling R. A., Klunk W. E. (2012). Brain imaging in Alzheimer disease. *Cold Spring Harbor Perspectives in Medicine*.

[6] Klunk W. E., Engler H., Nordberg A. (2004). Imaging brain amyloid in Alzheimer's disease with Pittsburgh Compound-B. *Annals of Neurology*.

[7] Mountz J. M., Laymon C. M., Cohen A. D. (2015). Comparison of qualitative and quantitative imaging characteristics of [^11^C]PiB and [^18^F]flutemetamol in normal control and Alzheimer's subjects. *NeuroImage: Clinical*.

[8] Hsiao I.-T., Huang C.-C., Hsieh C.-J. (2012). Correlation of early-phase 18F-florbetapir (AV-45/Amyvid) PET images to FDG images: Preliminary studies. *European Journal of Nuclear Medicine and Molecular Imaging*.

[9] Nelissen N., Van Laere K., Thurfjell L. (2009). Phase 1 study of the Pittsburgh compound B derivative ^18^F-flutemetamol in healthy volunteers and patients with probable Alzheimer disease. *Journal of Nuclear Medicine*.

[10] O'Keefe G. J., Saunder T. H., Ng S. (2009). Radiation dosimetry of *β*-amyloid tracers ^11^C-PiB and ^18^F-BAY94-9172. *Journal of Nuclear Medicine*.

[11] Poisnel G., Dhilly M., Moustié O. (2012). PET imaging with [^18^F]AV-45 in an APP/PS1-21 murine model of amyloid plaque deposition. *Neurobiology of Aging*.

[12] Rojas S., Herance J. R., Gispert J. D. (2013). In vivo evaluation of amyloid deposition and brain glucose metabolism of 5XFAD mice using positron emission tomography. *Neurobiology of Aging*.

[13] Von Reutern B., Grünecker B., Yousefi B. H., Henriksen G., Czisch M., Drzezga A. (2013). Voxel-based analysis of amyloid-burden measured with [^11^c]pib pet in a double transgenic mouse model of alzheimer's disease. *Molecular Imaging and Biology*.

[14] Snellman A., López-Picón F. R., Rokka J. (2013). Longitudinal amyloid imaging in mouse brain with 11C-PIB: Comparison of APP23, Tg2576, and APPswe-PS1dE9 mouse models of Alzheimer disease. *Journal of Nuclear Medicine*.

[15] Brendel M., Jaworska A., Grießinger E. (2015). Cross-sectional comparison of small animal [^18^F]-florbetaben amyloid-PET between transgenic AD mouse models. *PLoS ONE*.

[16] Lee B. S., Chu S. Y., Kwon H. R. (2016). Synthesis and evaluation of 6-(3-[^18^F]fluoro-2-hydroxypropyl)-substituted 2-pyridylbenzothiophenes and 2-pyridylbenzothiazoles as potential PET tracers for imaging A*β* plaques. *Bioorganic & Medicinal Chemistry*.

[17] Oh S. J., Kim M. H., Han S. J. (2017). Preliminary PET Study of ^18^F-FC119S in Normal and Alzheimer's Disease Models. *Molecular Pharmaceutics*.

[18] Byun B. H., Kim B. I., Park S. Y. (2017). Head-to-head comparison of ^11^C-PiB and ^18^F-FC119S for A*β* imaging in healthy subjects, mild cognitive impairment patients, and Alzheimer's disease patients. *Medicine (United States)*.

[19] Mullan M., Crawford F., Axelman K. (1992). A pathogenic mutation for probable Alzheimer's disease in the APP gene at the N–terminus of *β*–amyloid. *Nature Genetics*.

[20] Eckman C. B., Mehta N. D., Crook R. (1997). A new pathogenic mutation in the APP gene (1716V) increases the relative proportion of A*β*42(43). *Human Molecular Genetics*.

[21] Goate A., Chartier-Harlin M.-C., Mullan M. (1991). Segregation of a missense mutation in the amyloid precursor protein gene with familial Alzheimer's disease. *Nature*.

[22] Citron M., Eckman C. B., Diehl T. S. (1998). Additive effects of PS1 and APP mutations on secretion of the 42- residue amyloid *β*-protein. *Neurobiology of Disease*.

[23] Oakley H., Cole S. L., Logan S. (2006). Intraneuronal *β*-amyloid aggregates, neurodegeneration, and neuron loss in transgenic mice with five familial Alzheimer's disease mutations: potential factors in amyloid plaque formation. *The Journal of Neuroscience*.

[24] Macdonald I. R., DeBay D. R., Reid G. A. (2014). Early detection of cerebral glucose uptake changes in the 5XFAD mouse. *Current Alzheimer Research*.

[25] Schwarz C. G., Senjem M. L., Gunter J. L. (2017). Optimizing PiB-PET SUVR change-over-time measurement by a large-scale analysis of longitudinal reliability, plausibility, separability, and correlation with MMSE. *NeuroImage*.

[26] Logan J., Fowler J. S., Volkow N. D., Wang G.-J., Ding Y.-S., Alexoff D. L. (1996). Distribution volume ratios without blood sampling from graphical analysis of PET data. *Journal of Cerebral Blood Flow & Metabolism*.

[27] Matsubara K., Ibaraki M., Shimada H. (2016). Impact of spillover from white matter by partial volume effect on quantification of amyloid deposition with [11C]PiB PET. *NeuroImage*.

[28] Kimura R., Ohno M. (2009). Impairments in remote memory stabilization precede hippocampal synaptic and cognitive failures in 5XFAD Alzheimer mouse model. *Neurobiology of Disease*.

[29] McGowan E., Eriksen J., Hutton M. (2006). A decade of modeling Alzheimer's disease in transgenic mice. *Trends in Genetics*.

[30] Willuweit A., Velden J., Godemann R. (2009). Early-onset and robust amyloid pathology in a new homozygous mouse model of Alzheimer's disease. *PLoS ONE*.

[31] Reilly J. F., Games D., Rydel R. E. (2003). Amyloid deposition in the hippocampus and entorhinal cortex: Quantitative analysis of a transgenic mouse model. *Proceedings of the National Acadamy of Sciences of the United States of America*.

[32] Elder G. A., Gama Sosa M. A., de Gasperi R. (2010). Transgenic mouse models of Alzheimer's disease. *Mount Sinai Journal of Medicine*.

[33] Maeda J., Ji B., Irie T. (2007). Longitudinal, quantitative assessment of amyloid, neuroinflammation, and anti-amyloid treatment in a living mouse model of Alzheimer's disease enabled by positron emission tomography. *The Journal of Neuroscience*.

[34] Hall A. M., Roberson E. D. (2012). Mouse models of Alzheimer's disease. *Brain Research Bulletin*.

[35] Aboagye E. O., Price P. M., Jones T. (2001). In vivo pharmacokinetics and pharmacodynamics in drug development using positron-emission tomography. *Drug Discovery Therapy*.

