# Supplementary material for: Early Detection of Aβ Deposition in the 5xFAD Mouse by Amyloid PET
Source: Contrast Media Mol Imaging. 2018 Feb 28;2018:5272014. doi: 10.1155/2018/5272014 (PMC5851318; doi:10.1155/2018/5272014)
Supplement: Supplementary Materials — Figure S1: quantification of Aβ deposition in the cortex and hippocampus. Values are presented as the mean ± SD; n.d.: not detected. Figure S2: immunohistochemical staining of Aβ in the cerebellum of WT and 5xFAD mice. [file 5272014.f1.docx]

**Supplementary Information**

**Early detection of Aβ deposition in the 5xFAD mouse by amyloid PET**

Se Jong Oh^1,6^, Hae-June Lee^2^, Kyung Jun Kang^1^, Sang Jin Han^1^, Yong Jin Lee^1^, Kyo Chul Lee^1^, Sang Moo Lim,^5^ Dae Yoon Chi^3^, Kyeong Min Kim^4,6^, Ji-Ae Park^1,*^, Jae Yong Choi^1,*^

^1^Division of RI-Convergence Research, Korea Institute Radiological and Medical Sciences, Seoul, Korea;

^2^Division of Basic Radiation Bioscience, Korea Institute of Radiological and Medical Sciences, Seoul, Korea;

^3^Research Institute of Labeling, FutureChem Co., Ltd, Seoul, Korea;

^4^Division of Medical Radiation Equipment, Korea Institute Radiological and Medical Sciences, Seoul, Korea;

^5^Department of Nuclear Medicine, Korea Institute of Radiological & Medical Science, Seoul, Korea;

^6^Radiological & Medico-Oncological Sciences, University of Science and Technology, Daejeon, Korea.


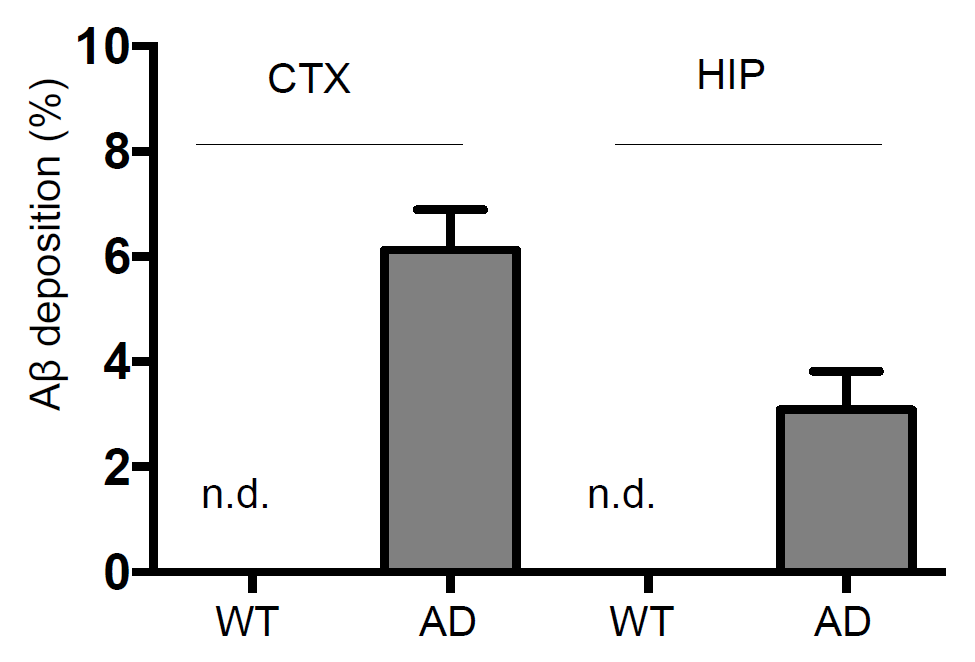


**Figure S1.** Quantification of Aβ deposition in the cortex and hippocampus. Values are presented as the mean ± SD, n.d.; not detected.


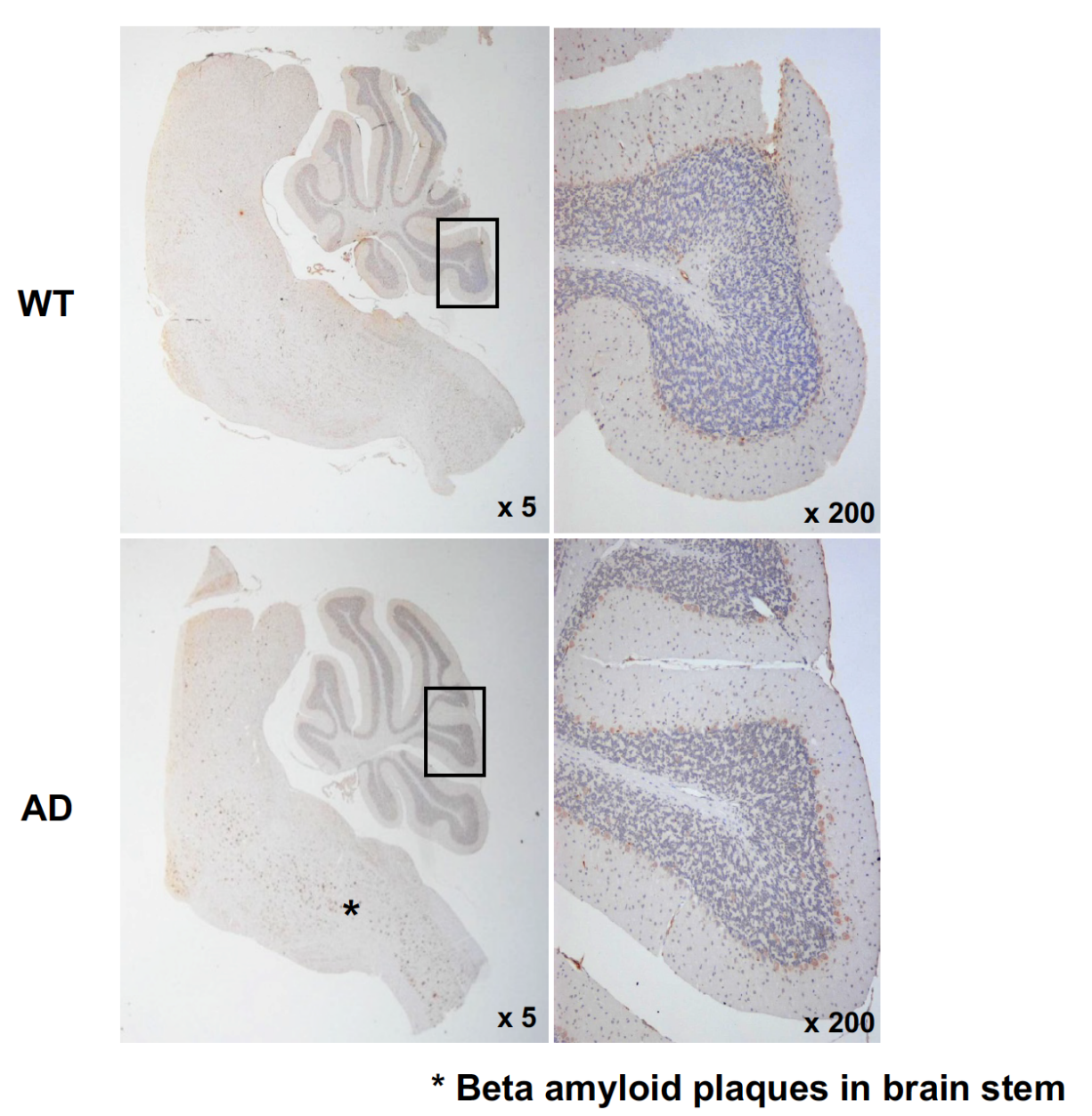


**Figure S2.** Immunohistochemical staining of Aβ in the cerebellum of WT and 5xFAD mice.
